# Supplementary material for: A comparison of control samples for ChIP-seq of histone modifications
Source: Front Genet. 2014 Sep 25;5:329. doi: 10.3389/fgene.2014.00329 (PMC4174756; doi:10.3389/fgene.2014.00329)
Supplement: Supplementary file 1 [file DataSheet1.DOCX]

# Supplementary methods





Supplementary figure 1: MA plots comparing enrichment between WCE, H3 and a randomly distributed sample over 1kbp bins across the genome. (A) WCE vs random, (B) H3 vs random (C) WCE vs H3 is provided on the same scale for comparison.

## MACS peaks with and without control

Running MACS on the H3K27me3 samples without a control yields 9565 peaks. Out of the new peaks introduced by adding the controls (peaks not overlapping peaks in the run without control), 2590 (WCE) and 3090 (H3) have a score above 109 (75% quantile of all peak scores with any control sample). Out of these, 1502 (WCE) and 1700 (H3) overlap a promoter region. The genes corresponding to these promoters are mainly unexpressed, as can be seen in supplementary figure 2. We speculate that these are genes with highly compressed DNA, giving few fragments in the H3K27me3 pull-down. Only by comparing to the even fewer fragments in the control samples do these peaks appear in the analysis.

Supplementary figure 2: Distribution of expression of genes with new peaks in the promoter from the controls (red line for H3 and blue line for WCE), compared to the distribution of all genes (grey bars). The coloured lines are increased by a factor 10 for easier comparison.


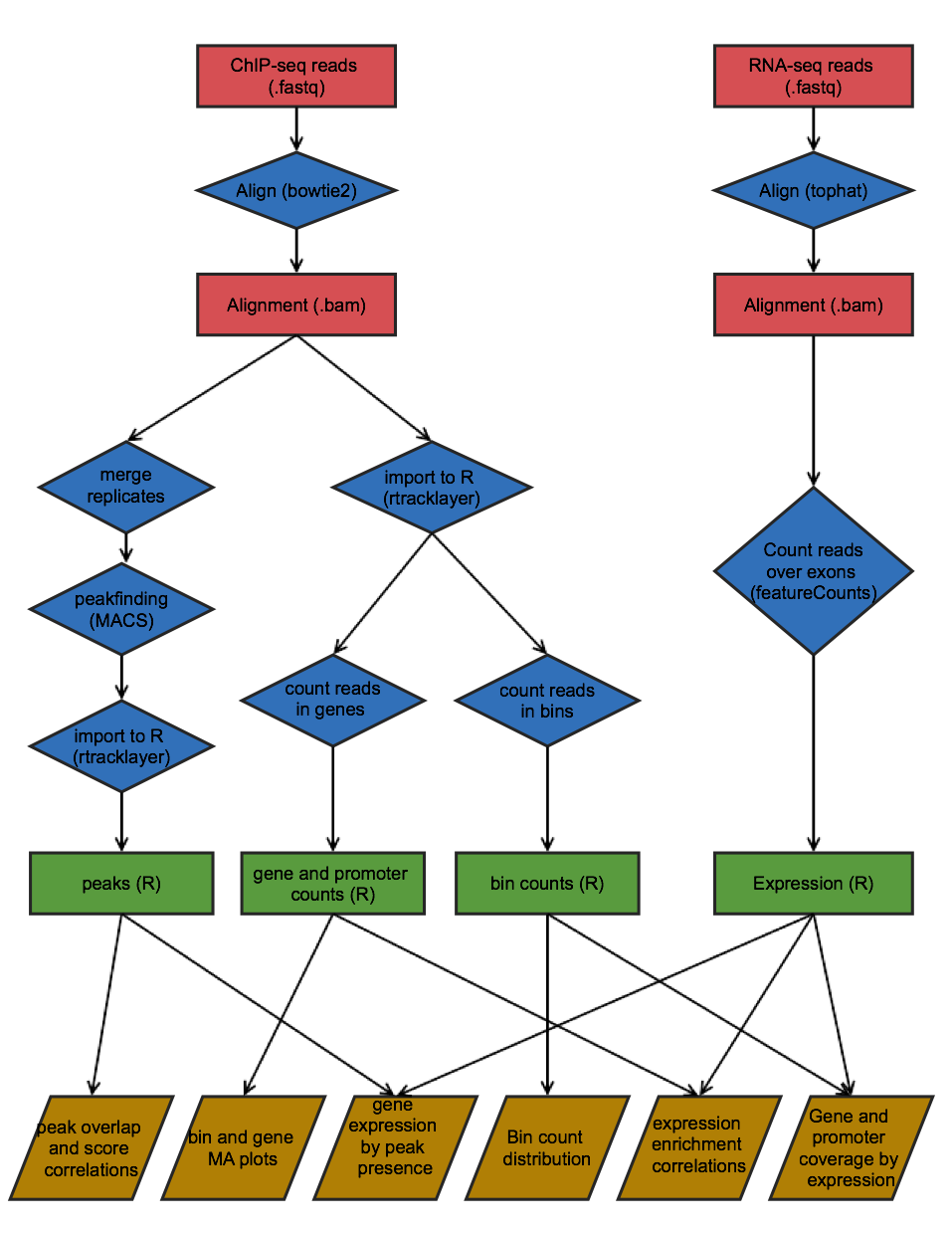


Figure 3: Workflow of analysis.
